# Fabrication and Characterization of Gliclazide Nanocrystals

**DOI:** 10.15171/apb.2018.049

**Published:** 2018-08-29

**Authors:** Nagaraju Ravouru, Rajeswari Surya Anusha Venna, Subhash Chandra Bose Penjuri, Saritha Damineni, Venkata Subbaiah Kotakadi, Srikanth Reddy Poreddy

**Affiliations:** ^1^Institute of Pharmaceutical Technology, Sri Padmavathi Mahila University, Tirupati, Andhra Pradesh, India.; ^2^Department of Pharmaceutics, MNR College of Pharmacy, Sangareddy, Telangana, India.; ^3^Department of Pharmaceutics, Sultan-ul-Uloom College of Pharmacy, Hyderabad, Telangana, India.; ^4^Research Scientist, DST Purse Centre, S.V. University, Tirupati, Andhra Pradesh, India.

**Keywords:** Dissolution rate, Gliclazide, Particle size, Sonoprecipitation, Zeta potential

## Abstract

***Purpose:*** The main aim of the present investigation was to enhance the solubility of poorly soluble Gliclazide by nanocrystallization.

***Methods:*** In present investigation gliclazide nanocrystals were prepared by sonoprecipitation using Pluronic F68, Poly Vinyl Alcohol (PVA), Poly ethylene Glycol 6000 (PEG), Poly Vinyl Pyrrolidine (PVP K30) and Sodium Lauryl Sulphate (SLS) as stabilizers. Fourier Transform Infrared Spectroscopic study (FTIR), Differential Scanning Calorimetry (DSC) and X ray diffraction (XRD) studies were conducted to study the drug interactions. Size and zeta potential of the nanocrystals were evaluated. In vitro and in vivo studies of nanocrystals were conducted in comparison to pure gliclazide.

***Results:*** The Gliclazide nanocrystals (GN) showed mean particle size of 131±7.7 nm with a zeta potential of -26.6 mV. Stable nanocrystals were formed with 0.5% of PEG 6000. FTIR, DSC and XRD studies of nanocrystals showed absence of interactions and polymorphism. SEM photographs showed a change in morphology of crystals from rod to irregular shape. There is an increase in the saturation solubility and the percentage drug release from formulation GN5 (Optimized Gliclazide Nanocrystals) was found to be 98.5 in 15 min. In the in vivo study, GN5 nanocrystals have reduced the blood glucose level to 296.4±4.26 mg/dl in 12 hr. The nanocrystals showed lower tmax and higher Cmax values as compared to pure gliclazide.

***Conclusion:*** The prepared nanocrystals of gliclazide were stable without any drug polymer interactions. Increase in the dissolution of nanocrystals compared to pure gliclazide and significant reduction in blood glucose level in vivo indicated better bioavailability of the nanocrystals. Therefore, it is concluded that nanocrystal technology can be a promising tool to improve solubility and hence dissolution of a hydrophobic drug.

## Introduction

According to Biopharmaceutical classification system drugs are categorized into 4 classes, each class having its own rate limiting factor affecting its bioavailability. At present most of the drugs (approximately 70%) in the development pipelines belongs to class II and have rate of dissolution as limiting factor for the absorption due to poor solubility.^1^

Though different approaches are available to improve the rate of dissolution, drug nanocrystals are preferred over the other. Nanocrystals are particles with nanometric dimension made from 100% drug; typically they are stabilized by surfactants or polymeric stabilizers.^2^ Nanocrystals also exhibit other advantages like increased saturation solubility and dissolution velocity which can be attributed to higher effective surface area and excellent adhesion to biological surfaces by which not only the bioavailability can be improved but the variations in the bioavailability can be reduced, an advantage which cannot be attained by microcrystals.^1,3^

Bottom-up process results in nanocrystals with small particle size and narrow size distribution compared to top-down process but have the main limitation of uncontrolled particle growth.^4^ Usage of an external ultrasonic wave force has been proved to make more intense mass transfer, increase in rate of molecular diffusion and lesser induction time of crystallization, enhancement in the nucleation rate, leading in reduction of crystal size, hindrance of agglomeration and regulation of the crystal size distribution.

Gliclazide is a 2^nd^ generation sulphonylurea, which shows less prevalence of hypoglycemia, low rate of secondary failure and has a potential for slowing the progression of diabetic retinopathy. Hence, gliclazide used as drug of choice in long term for the control of NIDDM. Gliclazide is a BCS class II drug having low aqueous solubility, which resulted in poor dissolution rate and intersubject variability of its bioavailability. So, in the present study nanocrystallization of gliclazide by sonoprecipitation method is employed to enhance the solubility, thereby to increase its bioavailability.^5^

## Materials and Methods

Gliclazide was kindly provided by Dr. Reddy’s laboratories, Hyderabad, India. Chemicals and reagents used were of analytical grade and were purchased from Merck, Mumbai, India.

### Preparation of gliclazide nanocrystals

Gliclazide nanocrystals were prepared by the combination of precipitation and ultrasonication (sonoprecipitation) using probe sonicator. Gliclazide was dissolved in DMSO to form an organic drug solution of concentration 30 mg/ml. Two ml of drug solution was added to the stabilizer solution (at a ratio of 1:20 v/v) fixed under the probe which was immersed 10 mm in the liquid which leads the wave transferring downwards and upwards (Hielscher Ultrasonics GmbH., Germany) at an ultrasonic power input of 200 W and an amplitude of 40% for 15 min. The ultrasonic sound burst was set to 0.5 sec with a pause of 0.5 sec between two bursts. During the preparation process the temperature was maintained by using an ice-water bath. The formed nanodispersion was concentrated by centrifugation at 15,000 rpm for 20 min using a cooling centrifuge (Remi Instruments ltd, India) and the product was vacuum filtered and air dried.^6^

### Particle size analysis

The average particle size of gliclazide nanocrystals was determined by light scattering technique (Nano Partica SZ-100-Z, Horiba Ltd., Japan). The dried nanocrystals were diluted with water before measurement and analyzed at scattering angle of 90°C.^7^

### Particle shape and morphology

The shape and morphology of gliclazide and optimized formulation was determined using scanning electron microscopy (SEM) (Zeiss EVO MA 15). Nanocrystals were placed on a glass slide and kept under vacuum. The nanocrystals were coated with a thin gold layer using a sputter coater unit. The scanning electron microscope was operated at 15 kV acceleration voltage.^7^

### Zeta potential

The zeta potential was measured by photon correlation spectroscopy instrument (Malvern, UK) at 25°C.^7^

### Fourier transform infrared spectroscopy

The FTIR spectral measurements were taken at ambient temperature using a Shimadzu, Model 8400 (USA). About 2 mg of the crude gliclazide and gliclazide nanocrystals were dispersed in KBr powder and the pellets were made by applying 6000 kg/cm^2^ pressure. FTIR spectra were obtained by powder diffuse reflectance on FTIR spectrophotometer.^7^

### Differential scanning calorimetry (DSC)

DSC studies were performed by using DSC-60 calorimeter (Schimadzu Corporation, Japan) to evaluate the molecular structure of crude gliclazide, and gliclazide nanocrystals.^7^

### X-Ray powder diffraction

The X-ray powder diffraction (XRPD) studies were carried by using a powder X-ray diffractometer (Rigaku Geigerflex XRD, Co., Japan). XRPD studies were measured at 40 kV and 45 mA. During XRPD studies the range of 2*θ* angle was used between 5° and 40° at a scan rate and step size of 5°/min and 0.01° respectively.^7^

### Saturation solubility

Saturated solution of gliclazide and gliclazide nanocrystals was prepared in 5 ml of water. Saturated solution was centrifuged at 17,000 rpm for 3 hr. Supernatant liquid was collected and the gliclazide concentration was analyzed at 228 nm by using UV spectrophotometer (Schimadzu Corporation, Japan).^8^

### Determination of residual solvents concentration

Gas chromatography (Shimadzu GC-14B Chromatograph, Japan) was used to estimate residual DMSO in gliclazide nanocrystals.^9^

### Dissolution studies

Dissolution studies of pure gliclazide and gliclazide nanocrystals was carried using USP XXIV-Type II (Electro Lab, Mumbai, India). 900 ml of 0.1 N HCl was used as dissolution medium. 5 ml samples were withdrawn at the end of 10, 20, 30, 40, 50 and 60 min and sink conditions were maintained. The gliclazide content in the samples was analyzed at 228 nm by using UV spectrophotometer (Schimadzu Corporation, Japan).^8,10^

### In vivo studies

#### Pharmacodynamic studies

The anti-diabetic activity of gliclazide nanocrystals was evaluated by alloxan-induced diabetic method. Study protocol was approved by IAEC (Reg. No. IAEC/1434/PO/E/S/11/2015). Male/female wistar rats weighing around 275-350 gm were used for the study. Rats were housed in polypropylene cages at a temperature 25±2°C with 12 hr day and 12 hr night cycle. All animals received standard laboratory diet and water ad libitum. Diabetes was induced in rats by administration of alloxan solution (150 mg/kg) intraperitonially. After a week, diabetic rats with fasting blood glucose of > 300 mg/dl were included in the study for 12 hr. These rats were divided into two groups of six rats in each and treated as below.

#### Animal grouping for anti-diabetic studies

The animals were divided into two groups (six animals in each group) for anti-diabetic studies.

Group I: Diabetes induced and gliclazide pure drug administered animals

Group II: Diabetes induced and gliclazide nano crystals administered animals 

Pure gliclazide and gliclazide nanocrystals (Dose: 2 mg/kg) was given by using oral feeding needle. Blood samples (0.1 ml) were collected through retro-orbital plexus under ether anesthesia at regular intervals for 12 hours. The blood glucose levels were determined using the commercial glucose kit. Comparative *in vivo* blood glucose level in alloxan-induced diabetic rats after oral administration of pure gliclazide and gliclazide nanocrystals were evaluated.^11,12^

#### Pharmacokinetic studies

Pharmacokinetic study protocol was approved by the IAEC (Reg. No. IAEC/1434/PO/E/S/11/2015). The oral pharmacokinetics of pure gliclazide and gliclazide nanocrystals was assessed in wistar rats. Animals were fasted overnight with free access to water for 12 hr before dosing. A dose equivalent to 2 mg/kg of pure gliclazide and gliclazide nanocrystals was given by using oral feeding needle. 0.1 ml of blood was collected from each rat through retro-orbital plexus under ether anesthesia at 1, 2, 4, 6, 8, 10 and 12 hr and the plasma was separated from blood by centrifuging at 2500 rpm for 10 min and stored under refrigerated conditions prior to assay on the same day. Assay of the gliclazide in plasma was estimated by HPLC method. The pharmacokinetic parameters were calculated using WinNonlin software.^13,14^

## Results and Discussion

### Preparation of gliclazide nanocrystals

Crystal formation includes particle nucleation, molecular growth of the particle and agglomeration/ aggregation of the particles, and their rate determines the particle size and size distribution.^4,5^ The driving force of the crystal formation is supersaturation, which determines the particle nucleation rate and also the diffusion-controlled growth rate of the crystal. The crystal growth depends on the rate of supersaturation, higher the rate of supersaturation leads to faster crystal growth.

### Solvent and anti solvent

Nucleation of a drug molecule is faster when it is dissolved in good solvent, because availability of more monomers in solvent phase for nucleation. So, DMSO was selected as a good solvent for gliclazide. In DMSO gliclazide showed nucleation at low supersaturation condition. In case of acetone and methanol the solubility of gliclazide is low which may not provide sufficient supersaturation.^4^ Water is selected as anti solvent due to the low solubility of gliclazide. Moreover, if the drug molecules having crystalline properties water is used as anti-solvent in most of the studies.^15^ DMSO: Water (1:20 ratio) was used to attain better particle size. With an increase in anti-solvent to solvent ratio the size of the crystal gradually decreases due to increase in the degree of supersaturation.^16,17^ At this antisolvent to solvent ratio (1:20), the nucleation rate is high and at the same time higher solvent phase instigate the particle growth, which leads to increase in the particle size.

### Effect of drug concentration

Gliclazide concentration in the solvent was selected to be 30 mg/ml as increase in concentration of the gliclazide may also lead to the increase in the particle size of the crystals. Higher the supersaturation, the faster will be the crystal growth which may be due to higher drug concentration, higher diffusion-controlled growth and higher agglomeration rate.^4,5^

### Effect of temperature

Temperature plays major role of particle size in crystallization. At a higher temperature, solubility of gliclazide increased which leads to lower rate of nucleation. Which may be due to reduction in the level of supersaturation. So, less crystal nuclei will be available for crystal growth and leads to formation of larger crystals.^18^ After nucleation, surface of the crystal will grow in a molecular way by two major pathways; 1. Movement of solute molecules from solution to the surface of the crystal diffusion, convection and combination of diffusion and convection. 2. Addition of molecules into the crystal lattice by surface integration (surface reaction process). At higher temperature faster crystal growth rate will occur due to higher diffusion and increased reaction kinetics at the interface. So, 3°C was selected as suitable crystallization temperature to get small particle with narrow particle size distribution.^4,5^

### Effect of ultrasonication 

Ultrasonication converts thermodynamically unstable matter into a more stable form by single/multiple application of energy, followed by thermal relaxation. Lowering of energy can be achieved by conversion of the solid from a less ordered to a more ordered lattice structure (an amorphous state to a crystal form). Alternatively, lowering of energy can be achieved by reducing the surface energy at the solid liquid interface by using surfactant molecules.^18^ During the crystallization process, ultrasound/sonication enhances the mixing and provides uniform conditions. Ultrasonication improves the mass transfer and enhances the molecular diffusion which leads to reduction in the crystallization time and enhancement in nucleation rate. This process helps to reduce the crystal size, agglomeration and narrow particle size distribution.^18^ Precipitation may occur in amorphous forms which were usually unstable and tends to transform into stable crystalline forms by the application of external energy.^18^

### Effect of stabilizer on particle size

Simple precipitation method has a limitation of uncontrolled nucleation crystal growth and spontaneous agglomeration, thus small particles need to be stabilized by surfactant or/and protective polymer. PEG 6000, Pluronic F68, PVPK30, PVA and SLS were screened as stabilizers for preparing gliclazide nanocrystals. The particle size and zeta potential are taken as basis for the selection of the best stabilizer. PEG 6000 allows the production of crystals with mean particle diameter of 128.6 nm and low particle size distribution of 7.7 nm (Table 1). Mean particle size of nanocrystals produced by all other stabilizers was beyond 128.

**Table 1:** Particle size distribution of gliclazide nanocrystals in different stabilizers

**Stabilizer**	**Particle size (nm)**	**Zeta Potential (mV)**
Pluronic F68	249.1 ± 28.7	-16.7
PVA	197.6 ± 18.6	-2.9
PEG 6000	128 ± 7.7	-26.6
PVPK 30	765.4 ± 27.6	-11.8
SLS	681.1 ± 19.3	-5.6

Mean ± SD, (n=3)

### Effect of stabilizer on zeta potential

Particle charge is one of the factors determining their physical stability. The higher is the electronic repulsion between the particles and the higher is the physical stability. As a rule of thumb, particles with zeta potential above 30 mV (absolute value) are physically stable. Particles with a potential above 60 mV show excellent stability. Particles below 20 mV are of limited stability; below 5 mV they undergo pronounced aggregation. In case of steric stabilizers zeta potential of about 20 mV was still sufficient to stabilize the system completely as the adsorption of a steric stabilizer layer leads to drop in the measured zeta potential, which is however not an indication of a reduced electrostatic repulsion. The adsorption layer of the steric stabilizer shifts the plane, at which the zeta potential is measured at away from the particle surface. Consequently, the measured zeta potential is lower. From the zeta potential analysis, it was found that PEG 6000 resulted in surface charge of -26.6 mV (Table 1) which is sufficient for stabilization of nanocrystals. Though PVA resulted in particle size nearer to the PEG 6000 formulation, they were found to have low zeta potential which is not sufficient enough for the stabilization of nanoparticles.

### Effect of stabilizer concentration

A suitable stabilizer concentration should be used for each drug to achieve smaller particle size. The crystal growth was shielded by the sorption of stabilizers. The amount/concentration of stabilizer should be abundant for full coverage of the crystal surface to provide adequate steric repulsion between the crystals. Inadequate surface coverage by stabilizer leads to rapid crystal growth and agglomeration. While high concentration of stabilizer will hinder the transmission of ultrasonic vibration and the diffusion between solvent and the anti-solvent during precipitation which may be due to enhanced viscosity. To optimize the minimum concentration of PEG 6000 which could produce smallest stable gliclazide nanocrystals possible with higher dissolution rate, the concentration of stabilizer is varied in the formulations (Table 2).

**Table 2:** Composition of different nanocrystal formulations

	**GN1**	**GN2**	**GN3**	**GN4**	**GN5**
Gliclazide (mg)	30	30	30	30	30
DMSO (ml)	1	1	1	1	1
PEG 6000 (%)	0.05	0.1	0.25	0.5	1
Water (ml)	20	20	20	20	20

### Particle shape and morphology

**Figure 1:**
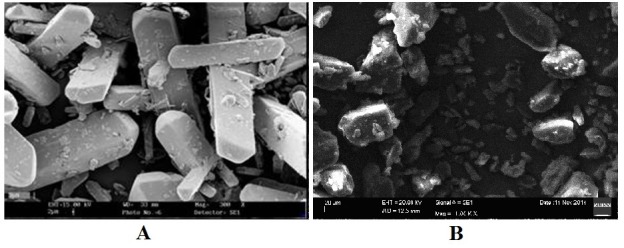
SEM images of A. Pure gliclazide B. Gliclazide nanocrystals

The morphology of nanocrystals formed by precipitation is altered by stabilizers used and drug concentration, so, SEM imaging was performed. From the images (Figure 1) it was found that nanocrystals are in irregular shape with uniform size while pure gliclazide was long cylindrical and non-uniform.

### Fourier transform infrared spectroscopy

**Figure 2:**
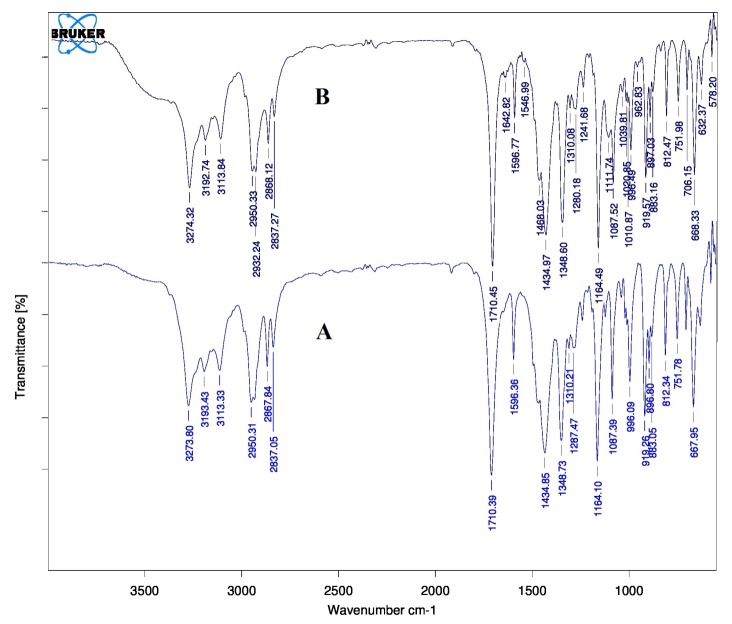
FTIR spectra of A. Pure gliclazide, B. Gliclazide nanocrystals

FTIR spectra showed characteristic peaks that belong to gliclazide in nanocrystals (Figure 2) without any shifting in bands indicating that chemical structure of gliclazide was preserved in the nanocrystal formulation and the preparation method employed or stabilizer had no effect on the stability of gliclazide. It further shows that there were no chemical interactions. The new bands found in the FTIR spectra of nanocrystals may be due to the stabilizer (PEG 6000).

### Differential scanning calorimetry

**Figure 3:**
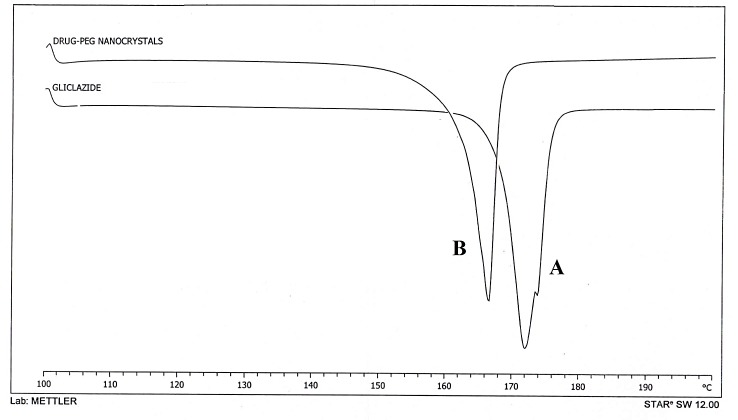
DSC thermograms of A. Pure gliclazide, B. Gliclazide nanocrystals

The gliclazide coarse powder exhibited a single endothermic peak at 172°C. The thermogram of nanocrystals showed characteristic endothermic peak corresponding to that of pure gliclazide with slight change in crystallinity due to change in melting point 167°C (Figure 3). No additional peaks are found in thermogram which indicates the absence of polymorphic changes in gliclazide nanocrystals.^19-21^

### X-ray powder diffraction (XRPD)

**Figure 4:**
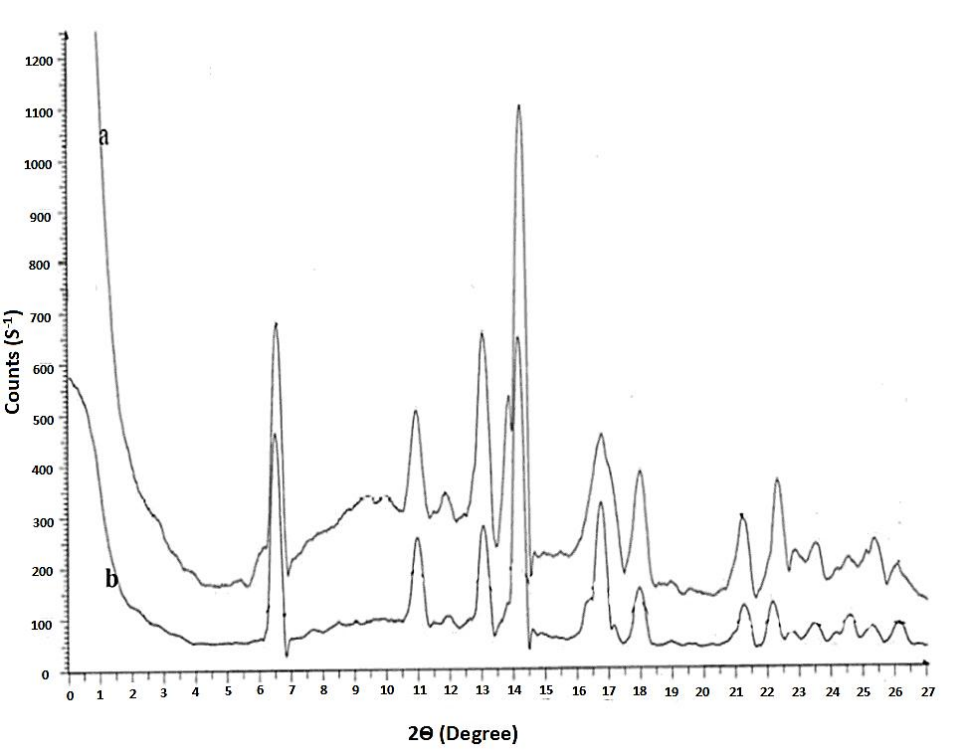
XRD spectra of A. Gliclazide pure drug, B. Gliclazide nanocrystals

During the precipitation process there is possibility of formation of amorphous or crystal compounds. In order to know the nature of the compound XRPD analysis was performed. As shown in the Figure 4, the XRPD pattern of the pure drug was very similar to that of the nanocrystalline compound, with characteristic peaks at diffraction angles between 10-11, 14.5-15.5, 16.5-19, 20.8-22.5, 25-26, 26.5-27.5, this indicate that crystallinity is retained in the nanocrystals with the same intensity as in gliclazide raw crystals. Conservation of the crystal structure/lattice of the drug in the formulation is very important for stability of the compound.

### Saturation solubility

Nanonisation has an extra effect when compared to micronisation. Nanonisation increases the surface area and saturation solubility of drugs. The solubility of drugs with normally depends on temperature and the solvent. The saturation solubility of a drug is a function of particle size. Due to strong curvature of the particles will enhance saturation solubility which may be due to increase in dissolution pressure. An increase in saturation solubility leads to enhancement of dissolution rate and formation of a supersaturated solution. This leads to increase in the concentration gradient between lumen of the gut and blood. This would enhance the drug diffusion from nanocrystals, promoting the better absorption. The saturation solubility of the formulations was determined with different concentrations of PEG 6000. It was found that there was an increase in the solubility of the gliclazide with an increase in the concentration of PEG 6000. The increase in solubility of the drug can be attributed both to the particle size and the stabilizer concentration (Table 3). The increased saturation solubility of the drug leads to an improvement in the solubility as well as bioavailability.^7^

**Table 3:** Saturation solubility data of pure gliclazide and nanocrystals

**Formulation**	**Solubility (gm/lit)**
Pure Gliclazide	0.022 ± 0.03
GN1	0.4 ± 0.01
GN2	0.512 ± 0.08
GN3	0.69 ± 0.3
GN4	0.75 ± 0.83
GN5	0.8 ± 0.5

Mean ± SD, (n=3)

### Residual solvents concentration 

The concentration of DMSO was found to be 791 ppm. According to guidelines for residual solvents Q3C (ICH), DMSO is class 3 solvent (solvent with low toxic potential), thus the limits of 5000 ppm is acceptable without justification.

### Dissolution studies

**Figure 5:**
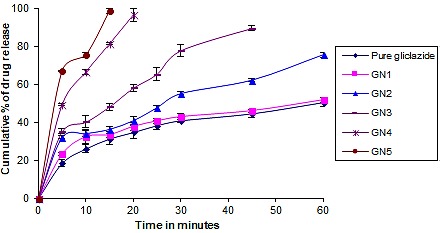
Dissolution profiles of pure gliclazide and gliclazide nanocrystals

The percentage release of gliclazide from pure drug was only 31.31±2.89 in 15 min while GN4 and GN5 showed 81.25±3.22 and 98.5±1.35 in 15 min (Figure 5) respectively which are having a high concentration of stabilizer. An increase in the concentration of stabilizer helped in better stabilization of nanocrystals and also an enhancement in dissolution of the drug was observed. Rate of dissolution was found to be higher for the nanocrystals produced with stabilizer concentration of 0.5% and 1% (GN4 & GN5). GN1, GN2 and GN3 (stabilizer concentration less than 0.5%) showed less improvement in dissolution rate which can be attributed to inefficient stabilization of nanocrystals and crystal growth resulting in high particle size. The rate of dissolution of nanocrystals (GN5) was found to be increased 3 times in comparison to the gliclazide raw crystals. Gliclazide nanocrystals (GN5) showed faster release so it was selected for further *in vivo* studies.

### In vivo studies

**Figure 6:**
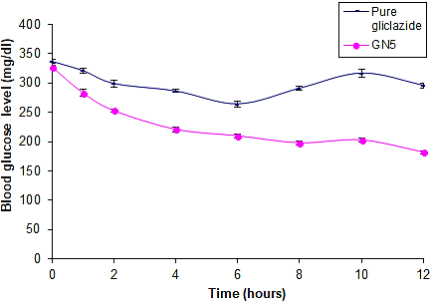
Comparative *in vivo* blood glucose level in diabetic rats after oral administration of pure gliclazide and GN5

Pharmacodynamic study was performed in diabetes induced rats. In diabetic rats, it was found that pure gliclazide and GN5 have reduced the blood glucose level to 296.4±4.26 mg/dl and 182.31±2.52 mg/dl respectively at the end of 12 hours (Figure 6). The decrease in glucose levels reflects an increase of gliclazide in the blood levels as a result of the drug dissolution and absorption.^13^

The absorption of gliclazide from nanocrystals was faster indicated by its low t_max_ value (2.12±3.29 hours) in comparison with pure gliclazide which may be due to increased solubility. The C_max_ value of gliclazide nanocrystals was comparatively higher than the pure gliclazide which might be due to better absorption. The t_1/2_ and MRT values of the gliclazide from powder and nanocrystals were found to be approximately 6 hours. This suggests that the drug stays in the body for a longer duration of time before being eliminated. AUC of gliclazide from nanocrystals was found to be significantly higher than the pure gliclazide (Table 4) which indicates an improvement in bioavailability of gliclazide from nanocrystals.

**Table 4:** Pharmacokinetic parameters of gliclazide in normal rats

**Parameter**	**Oral administration**	
	**Pure gliclazide**	**Gliclazide nanocrystals (GN5)**
C_max_ (µg/ml)	34.78±3.22	50.52±4.33
t_max_ (hr)	2.7±4.58	2.12±3.29
t_1/2_ (hr)	6.79±2.24	6.18±3.80
MRT (hr)	6.12±1.24	6.73±1.80
AUC_0→ ∞_(hr.µg/ml)	217.24± 63.19	392.76±73.38

Mean ± SD, (n=6)

## Conclusion

Gliclazide nanocrystals formulated by sonoprecipitation showed enhanced dissolution rate. Fourier Infrared Spectroscopy (FTIR), Differential Scanning Calorimetry (DSC) and X ray diffraction (XRD) studies showed that there is no polymorphism or no change in the crystal structure. The gliclazide nanocrystals showed better antidiabetic activity in the rats compared to pure gliclazide due to enhanced solubility and absorption. MRT and AUC of gliclazide nanocrystals were found to be higher than the pure gliclazide which indicates an improvement in bioavailability of gliclazide from nanocrystals.

## Acknowledgments

The authors would like to thank Dr. Reddy’s laboratories, Hyderabad, India for providing gift sample of gliclazide.

## Ethical Issues

The protocol of animal use was in compliance with Committee for the Purpose of Control and Supervision of Experiments on Animals, India.

## Conflict of Interest

The authors declare no conflict of interests.
